# Post-exposure human rabies prophylaxis: spatial patterns of inadequate procedures in Ceará - Brazil, 2007 to 2015

**DOI:** 10.1590/0037-8682-0247-2019

**Published:** 2019-12-20

**Authors:** Kellyn Kessiene de Sousa Cavalcante, Caroline Mary Gurgel Dias Florêncio, Jarier de Oliveira Moreno, Francisco Gustavo Silveira Correia, Carlos Henrique Alencar

**Affiliations:** 1 Universidade Federal do Ceará, Faculdade de Medicina, Programa de Pós Graduação em Saúde Pública, Fortaleza, CE, Brasil.

**Keywords:** Post-exposure prophylaxis, Rabies, Spatial distribution

## Abstract

**INTRODUCTION:**

This study investigated the spatial distribution of inappropriate post-exposure human rabies procedures in Ceará, Brazil, between 2007 and 2015.

**METHODS:**

The ecological study population was based on the records of post-exposure human rabies procedures from the Notification Disease Information System. We analyzed the data using the Moran Index (I) and the Moran Local Index.

**RESULTS:**

There were 222,036 (95.8%) records with inappropriate post-exposure human rabies procedures. There was heterogeneity in their spatial distribution, with two significant clusters in the northeast and northwest regions.

**CONCLUSIONS:**

These findings help elaborate differentiated strategies to reduce unnecessary post-exposure human rabies procedures.

Human rabies is an acute viral infectious disease that remains a public health concern due to its high lethality^1^. This disease can be controlled and eliminated in urban environments through prevention, surveillance, and controlled actions directed towards human beings and animals^2^. The Brazilian Rabies Control Program is based on pre-exposure prevention efforts and post-exposure treatment. The treatment protocol involves a simple exposure-site wash with soap and water until the anti-rabies serum and vaccine can be administered^3^. The administration of post-exposure prophylaxis occurs after a detailed anamnesis^4^; however, unnecessary treatments burden public health resources, as can cause adverse events in patients^5^.

Over the last ten years, the amount of anti-rabies post-exposure treatment administered has doubled. Meanwhile, the type and severity of exposure to the rabies virus has been overestimated by Brazilian health authorities, resulting in an increase in treatments irrespective of its necessity^6^.

The human rabies treatment has been one of the most prevalent compulsory notifications in Ceará recently: 231.694 cases between 2007 and 2015 (an average of 29.702 cases per year)^5^. However, the real epidemiology of human rabies in Brazil is clouded by ineffective epidemiological surveillance actions and inadequate records. In this context, spatial analysis has emerged as an important tool to characterize the exposure to the disease and its risk factors^7^ ^,^ ^8^. 

Considering the importance of the epidemiology of rabies prophylaxis, this study aimed to characterize the spatial distribution of inappropriate post-exposure human rabies procedures from 2007 to 2015 in Ceará, Brazil.

An ecological study was carried out involving 184 municipalities in the state of Ceará between 2007 and 2015. With a population of approximately 9 million and an area of 149 thousand km² in the northeast region of Brazil, Ceará is bounded by the Atlantic Ocean to the north, the state of Piauí to the west, the states of Rio Grande do Norte and Paraíba to the east, and the state of Pernambuco to the south. It is divided into five macro-regions of health, namely: the Metropolitan region of Fortaleza, the Northwest region (Sobral), the South region (Cariri), the Central region (Central Sertão), and the Eastern region (Litoral Leste) (Figure 1).

**FIGURE 1:**
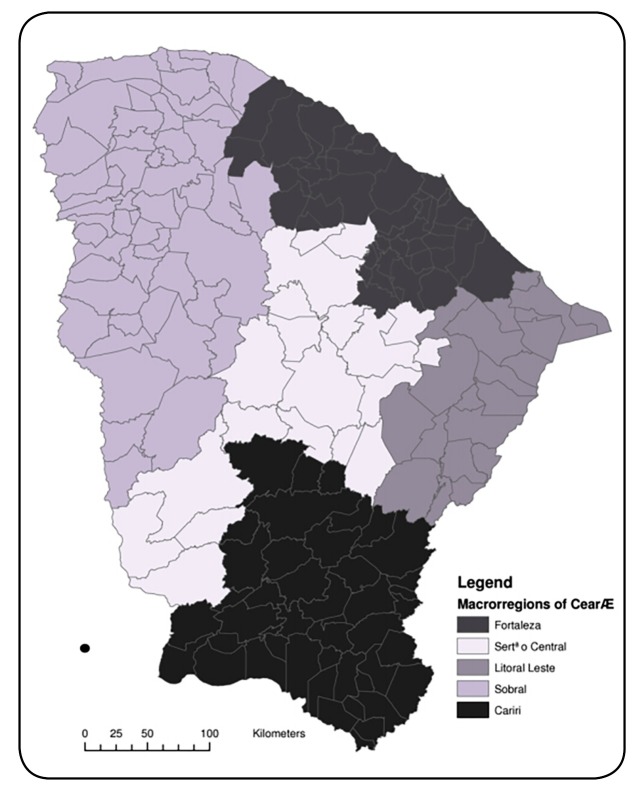
Map of the territorial distribution of the state of Ceará by macro-region of health.

The study population was based on the records of post-exposure human rabies extracted from the Notification Disease Information System (SINAN in Portuguese) of Ceará’s health department. The variables used to define the outcomes of this study were: improper use of vaccine/serum; number of doses not compatible with official treatments according to the characteristics of the exposure; or insufficient data (blank/ignored variables or factors disagreeing with the protocol).

To define the outcome variables, the terms “light procedure” and “severe procedure” were created based on the *Brazilian Technical Norms for Prophylaxis of Human Rabies*. The light procedure is appropriate when there are superficial wounds (usually only one) on the body or limbs. The severe procedure is appropriate when the wounds are located on the head, face, neck, hands, digital pulp and/or sole. Additionally, the severe procedure is also performed for deep, multiple, or extensive wounds in any region of the body, mucosal licking, or deep injury caused by animal nail. When evaluating the prophylactic procedures adopted in each situation, the term ‘adequate procedure’ was applied when the classification of light or severe procedures occurred based on their appropriate characteristics. The classifications considered the following variables: the type, location and depth of the lesion; type of exposure; species and condition of the aggressor animal; and use of vaccine treatment. Procedures that did not follow the protocol of the Brazilian Ministry of Health, or presented incomplete data were found to be inadequate, either due to an absence or an excess of treatment^3^ ^,^ ^5^ ^,^ ^9^. 

The geographical units of analysis were the 184 municipalities of Ceará. In order to reduce random variations, the average rates of inadequate post-exposure human rabies procedures were calculated and divided into blocks of time. They were stratified as 2007-2010, 2011-2013, and 2014-2015, as well as for the whole period of 2007-2015. These periods were divided according to the temporal trends of the rates of inadequate post-exposure human rabies procedures^10^. These rates were standardized by age group using a direct method. The estimated population for each year was based on the Brazilian Institute of Geography and Statistics data (IBGE). The standard reference population was taken from the Brazilian census of 2010. Georeferenced cartographic data were obtained from the National Institute of Space Research (INPE in Portuguese). 

Spatial autocorrelation of human rabies inadequate procedures incidence was measured using the Moran Index (I) and the Moran Local Index of Spatial Association (LISA) to identify the occurrence of clusters. We considered high risk areas to be a group of high incidence municipalities. 

The TerraView 4.2.2 software was used for processing and analyzing cartographic data and processing spatial autocorrelation indicators. Thematic maps were generated by ArcGis 9.2 software (Environmental Systems Research Institute - ESRI, Redlands, CA, USA).

The study was accepted by the Research Ethics Committee of the Federal University of Ceará under CAAE No. 64830316.0.0000.5054 and was carried out in accordance with the principles of Resolution 466/2012 of the National Health Council.

All 184 municipalities of Ceará register post-exposure human anti-rabies services. From 2007 to 2015, 231,694 incidents were reported involving the potential transmission of rabies in the state of Ceará. Of this total, there were 222,036 (95.8%) inadequate procedures deviating from the anti-rabies prophylactic treatment recommended by the Ministry of Health^9^.

The higher rates of inadequate post-exposure human rabies procedures ranged from 222.3 to 104.7 inadequate procedures per 100,000 inhabitants; the municipalities of Guaramiranga and Jijoca de Jericoacoara had particularly high number of inadequate procedures (222.3 and 131.5 per 100,000 inhabitants, respectively). We identified 23 municipalities (12.5%) with coefficients between 60.1 and 90.0, 77 (41.8%) from 30.1 to 60.0, and 76 (41.3%) from 0.1 to 30.0 inadequate procedures per 100,000 inhabitants. Only three municipalities did not record inadequate procedures (Figure 2).

Significantly higher values were observed in two high-high clusters, involving nine municipalities in the northeast of the state and four municipalities in the northwest region with rates of inadequate post-exposure human rabies procedures above 50 inadequate procedures per 100,000 inhabitants (Figure 2).

Between 2007 and 2015, seven (3.8%) municipalities in the central and southern regions of Ceará were identified as low-low clusters (less than 7 inadequate procedures per 100,000 inhabitants) (Figure 2).

**FIGURE 2:**
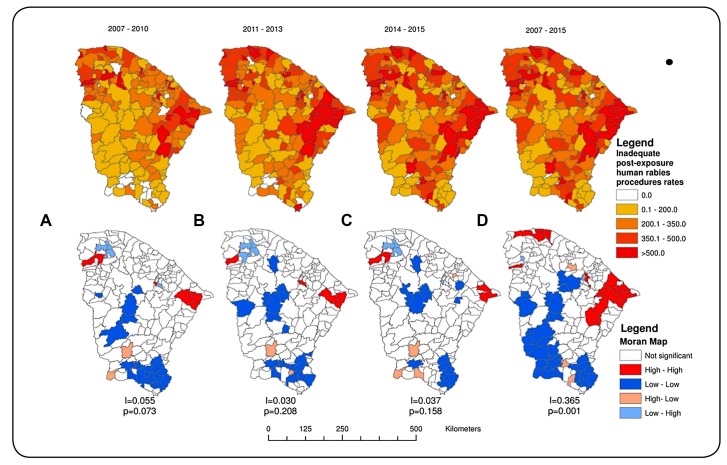
Spatial distribution and Moran Map of incidence coefficients (per 100000 population of inadequate conducts of post-exposure human anti-rabies services in Ceará, **(A)** 2007-2010, **(B)** 2011-2013, **(C)** 2014-2015 and **(D)** 2007-2015.

The spatial analysis showed geographic heterogeneity in the incidence and identified two well-defined spatial clusters of inadequate post-exposure human rabies procedures in the state of Ceará. Clusters of high rates of inadequate post-exposure human rabies procedures were identified in the northeast and northwest regions and low incidence clusters were identified in the south region. 

There were 62,642 (27.0%) records with ignored or blank variables between 2007 and 2015. This information plays an important role in health policy planning and decision making by providing epidemiological evidence for the detection of the population's health needs. In this way, the absence of information should be considered as an inadequate procedure in human anti-rabies prophylaxis^9^.

It is important to note the importance of completeness and consistency when filling in the forms to report accidents caused by animals that could potentially transmit rabies. These forms lead to the indication of the most appropriate prophylactic procedures for each case. In cases of serious injuries, considering the high risk of exposure to rabies, health care providers should administer the correct prophylaxis protocol, prescribed and monitored according to the norms of the Ministry of Health^5^.

These findings help develop the actions of the State Rabies Control Program, identifying areas with higher rates of inadequate post-exposure human rabies procedures. Further, they can elaborate differentiated strategies to control the disease in regions still at risk^11^. 

The higher rates of inadequate post-exposure human rabies procedures in Ceará are higher than the rates found in a study focusing on a municipality of Pernambuco state^11^. The correct and complete treatment, according to the type of exposure, must be guaranteed. If it is not completed according to the Brazilian Ministry of Health rules, the local control program must be reevaluated^5^ ^,^ ^9^. The assessments of the treatments must consider the type of exposure, injury and condition of the aggressor animal^3^ ^,^ ^5^. High-risk municipality clusters may be produced by structural deficiencies: high turnover of health professionals, absence of related education, lack of animal control, or administration of vaccines in immunosuppressed animals^12^. 

The high rates in urban areas of the north-eastern and north-western regions of Ceará reflect a social vulnerability. Namely, the disordered urbanization process favors the transmission of human rabies and the large number of domestic animals can cause potential sanitation or epidemiological threats^12^. 

In a ten-year study of the metropolitan region of São Paulo, the spatial pattern of human rabies procedures revealed that there was no record of animal aggressions in 24.6% of all neighborhoods. Further, there was a distinct distribution of animal aggressions between the neighborhoods^13^. 

In a small area, inadequate social conditions may favor the spread of the virus. Local social inequality increases contact between humans and domestic animals; this leads to poor health outcomes^14^. Environmental degradation related to uncontrolled domestic animals becomes a great risk to public health. This contributes to an increase in the number of diseases related to domestic animals and the incidence of zoonosis.

The rabies-control actions towards dogs and cats in urban areas of Ceará involve vaccination campaigns, coordinated by the State Department of Health, following the recommendations of the Ministry of Health. In 2015, the vaccination coverage of dogs was 101.8% (1,139,437 animals)^15^. However, Ceará is considered a high-risk area for human rabies due to the heterogeneity of the canine population vaccinations among municipalities, besides the presence of many unrestricted dogs in urban places. We highlight the importance of the domestic animals’ annual vaccination with a minimum of 80% vaccination coverage for dogs to prevent the virus from spreading^6^. Municipalities in the northeastern and northwestern regions of Ceará had a high number of inadequate procedures. However, in 2015, they achieved anti-rabies vaccine coverage rates above those recommended by the Brazilian Ministry of Health, with values ranging from 99.1 to 109.6^15^. 

There was an increasing number of inadequate behaviors reports for rabies treatments and of human rabies vaccines administered in Ceará. It is clear that intervention measures are needed, especially in these high risk areas of the state. 

This research manipulated secondary databanks, including notification forms with fields containing missing or incomplete data, and records considered inadequate. However, these deficiencies did not lead to the loss of information. 

Municipalities with the highest number of notifications aligned with areas of medium to high socioeconomic development. The spatial analysis identified that the geographical distribution of the misconduct of post-exposure human rabies procedures in Ceará is heterogeneous. The northeast and northwest regions were identified as higher risk areas; enhancement of rabies control interventions at the state and at municipal levels is a priority.

## References

[1] Ilyas N, Rahim K, Latif Z (2017). Incidence of dog bite in rural area (Chountra), District
Rawalpindi, Province Punjab, Pakistan. J. Nucl. Med. Allied Sci..

[2] Filgueira AdC, Cardoso MD, Ferreira LOC (2011). Profilaxia antirrábica humana: uma análise exploratória dos
atendimentos ocorridos em Salgueiro-PE, no ano de 2007. Epidemiol. Serv. Saúde.

[3] Ministério da Saúde (MS). Secretaria de Vigilância em Saúde.
Departamento de Vigilância Epidemiológica (2014). Normas técnicas de profilaxia da raiva humana.

[4] Veloso RD, Castro Aerts DRG, Fetzer LO, Anjos CB, Sangiovanni JC (2011). Epidemiologic profile of human anti-rabies treatment in Porto
Alegre, RS, Brazil. Ciênc. Saúde Colet.

[5] Cavalcante KK, Florêncio CM, Alencar CH (2017). Profilaxia antirrábica humana pós-exposição: características dos
atendimentos no estado do Ceará, 2007-2015. J. Health Biol. Sci..

[6] Bocchi MR (2017). Campanha antirrábica canina e felina: a importância da equipe de
trabalho: recursos utilizados e resultados obtidos pelos municípios no
desenvolvimento da campanha antirrábica canina e felina na região de São
José do Rio Preto/SP, Brasil, no período de 2009 a 2013. Rev. Educ. Cont. em Med. Veterin. e Zoot.

[7] Mascarenhas MTVL, Bahia Cerqueira R, Lobato Cardim L, Borio dos Santos Calmon Bittencourt TC, Peneluc T, Silva de Brito V (2012). Análise espacial dos dados do programa de profilaxia da raiva no
município de Lauro de Freitas, Bahia, Brasil, no período de
1999-2004. Rev. Baiana Saúde Pública.

[8] Barcellos CdC, Ramalho WM (2002). Situação atual do geoprocessamento e da análise de dados
espaciais em saúde no Brasil. 2002. Rev. Inform. Pública.

[9] Cavalcante KKdS, Alencar CH (2018). Raiva humana: avaliação da prevalência das condutas profiláticas
pós-exposição no Ceará, Brasil, 2007-2015. Epidemiol. Serv. Saúde.

[10] Cavalcante KKdS, Florêncio CMG, Alencar CH (2019). Atendimentos antirrábicos humanos pós-exposição: tendência
temporal de sua prevalência no Ceará, de 2007 a 2015. Cad. Saúde Colet..

[11] Silva FL, Ribeiro CRL, Coelho JMM, Sousa MEL, de Jesus Nascimento S, Batalha MA (2016). Ampliação do acesso ao atendimento antirrábico humano em São
Luís, Maranhão: Relato de experiência. Rev. Bras. Pesqui. Saúde.

[12] Santa Cruz L, Irene D, Hurtado Gascón L, Montalvo Reynoso Y, Varona Dávila S, Rodríguez Cruz J (2017). Comportamiento de los focos rábicos en la provincia La
Habana. Arch. Méd. Camaguey.

[13] Garcia RC, Vasconcellos SA, Sakamoto SM, Lopez AC (1999). Análise de tratamento anti-rábico humano pós-exposição em região
da Grande São Paulo, Brasil. Rev. Saúde Pública.

[14] Frias DFR, Nunes JOR, Carvalho AAB (2016). Proposta de nova metodologia de apoio para indicação racional de
profilaxia antirrábica humana pós-exposição. Arq. Ciências Saúde UNIPAR.

[15] Ministério da Saúde (MS). Secretaria de Vigilância em Saúde.
Departamento de Imunizações (2015). Sistema de Informação do Programa Nacional de Imunizações.

